# Serine Protease Inhibitor Attenuates Ovalbumin Induced Inflammation in Mouse Model of Allergic Airway Disease

**DOI:** 10.1371/journal.pone.0041107

**Published:** 2012-07-19

**Authors:** Sanjay Saw, Sagar Laxman Kale, Naveen Arora

**Affiliations:** Institute of Genomics and Integrative Biology, Delhi University Campus, Delhi, India; Albany Medical College, United States of America

## Abstract

**Background:**

Serine proteases promote inflammation and tissue remodeling by activating proteinase-activated receptors, urokinase, metalloproteinases and angiotensin. In the present study, 4-(2-Aminoethyl) benzenesulfonyl fluoride (AEBSF) a serine protease inhibitor was evaluated for prophylactic and therapeutic treatment in mouse model of airway allergy.

**Methods:**

BALB/c mice were sensitized by *i.p* route and challenged with ovalbumin. They were treated *i.n.* with 2, 10 and 50 µg of AEBSF, one hour before or after challenge and euthanized to collect BALF (bronchoalveolar lavage fluid), blood and lungs. Proteolytic activity, total cell/eosinophil/neutrophil count eosinophil peroxidase activity (EPO), IL-4, IL-5, IL-10, IL-13, cysteinyl leukotrienes and 8-isoprostane were determined in BALF and immunoglobulins were measured in serum. H&E and PAS stained lung sections were examined for cellular infiltration and airway inflammation.

**Results:**

Mice exposed to ovalbumin and treated with PBS showed increased cellular infiltration in lungs and higher serum IgE, IgG1 and IgG2a levels as compared to sham mice. Treatment with AEBSF reduced total cells/eosinophil/neutrophil infiltration. Both prophylactic and therapeutic AEBSF treatment of 10 or 50 µg reduced serum IgE and IgG1 significantly (p<0.05) than control. AEBSF treatment reduced the proteolytic activity in BALF. IL-4 IL-5 and IL-13 levels decreased significantly (p<0.05) after AEBSF treatment while IL-10 levels increased significantly (p<0.05) in BALF. Airway inflammation and goblet cell hyperplasia reduced as demonstrated by lung histopathology, EPO activity and cysteinyl leukotrienes in BALF after treatment. AEBSF treatment also suppressed oxidative stress in terms of 8-isoprostane in BALF. Among the treatment doses, 10 or 50 µg of AEBSF were most effective in reducing the inflammatory parameters.

**Conclusions:**

Prophylactic and therapeutic treatment with serine protease inhibitor attenuates the airway inflammation in mouse model of airway allergy and have potential for adjunct therapy.

## Introduction</emph>

Proteases are an important group of proteins implicated in manifestation of coagulopathies, respiratory inflammatory diseases, cancer and degenerative diseases [1]–[3]. Evidence shows that both intrinsic and extrinsic proteases play a major role in pathophysiology of airway diseases like asthma [4]. Proteolytic activity of allergens from fungi, pollens, animals, house dust mites and cockroaches augment allergic responses [5]–[9]. Furthermore, intrinsic proteases like mast cell tryptase initiates late phase allergic reactions [10].

Proteases exacerbate allergic diseases by compromising bronchial epithelial permeability [11], [12], disturbing protease antiprotease balance at lung surfaces, mediating cytokine release, activating PAR-2 receptors expressed by a variety of immune cells [13] and orchestrating Th-2 responses by cleaving CD23 on B-cells and CD25 on T-cells. Inactivated protease allergens have lesser potential in manifestation of allergic immune response [8]. Recently Post et al. [14] suggested that the epithelial barrier function of allergen is independent of protease activity. Targeting proteolytic activity by inhibitors can prove crucial to reduce proteases induced inflammatory diseases. Aprotinin prevented trypsin induced shock in dogs [15], chymase inhibitors SUN-C8257 [16], Y-40613 [17], and SUN-8077 [18], have shown to reduce dermatitis in animal models. AEBSF is an irreversible serine protease inhibitor with broad specificity (Trypsin, chymotrypsin, plasmin, thrombin, kallikreins) and high affinity. It inactivates the enzymes under acidic inflammatory condition, is non toxic (LD50 of 76 mg/kg), soluble in water (200 mg/ml) and excreted from the body. AEBSF is a unique molecule that can inhibit serine proteases as well as NADPH oxidase, a primary enzyme responsible for catalyzing production of ROS in epithelial cells, inflammatory cells and phagocytes [19]. Owing to these properties we hypothesized that AEBSF may reduce allergic airway inflammation. Current strategies for treatment of allergic diseases rely heavily on antihistamines and anti-inflammatory agents. The present study is therefore aimed to explore prophylactic and therapeutic effects of AEBSF in mouse model of allergic airway disease.

## Results

### AEBSF Treatment Reduces Cellular Infiltration in Lung

**Figure 1:**
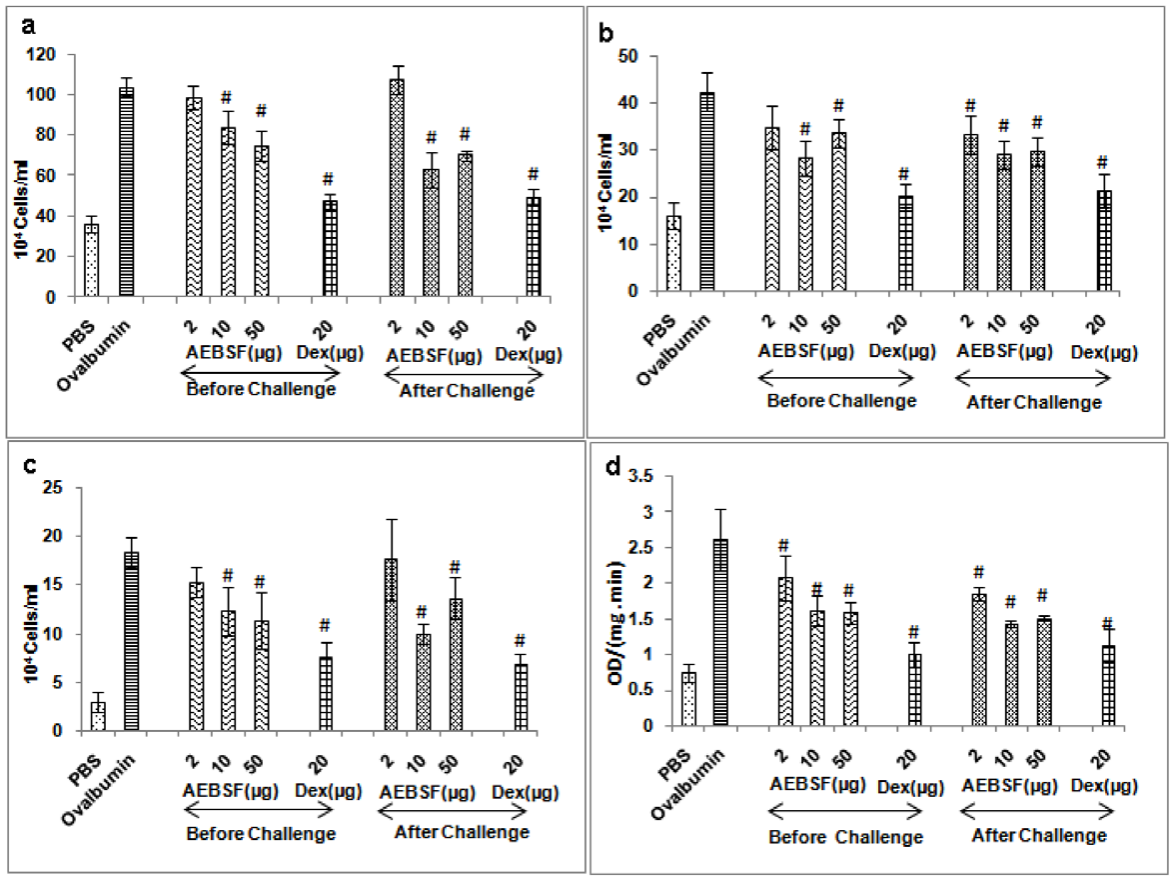
AEBSF/Dex (dexamethasone) treatment reduces cellular infiltration. (a) Total cell count (b) neutrophil count (c) eosinophil count and (d) EPO activity in terms of OD/(mg. min) in BALF of mice. Data are presented as mean ± SD of 6 mice per group for one of the two independent mice experiments. #: p<0.05.

Mice sensitized and challenged with ovalbumin have increased infiltration of inflammatory cells in BALF as compared to sham mice. AEBSF treatment significantly reduced the cellular infiltration in the lungs of mice compared to ovalbumin group (p<0.05). AEBSF treatment before antigen challenge reduced the total cells/eosinophil/neutrophil counts in a dose dependent manner (2, 10 and 50 µg) whereas the treatment after the challenge decreased maximum infiltration at 10 µg of AEBSF (Figure 1a-c). Dexamethasone also showed significant reduction in the total cells/eosinophil/neutrophil counts in both the treatment conditions in mice.

EPO in BALF of each treatment group was determined by ELISA (Figure 1d). AEBSF treatment reduced EPO activity in a dose dependent manner in both the treatment groups; however reduction was maximum at 10 µg of AEBSF for after challenge group of mice. The reduction in EPO activity was significant in both the treatment groups at all dose of AEBSF compared to ovalbumin group. Dexamethasone treatment also reduced the EPO activity significantly in BALF (p<0.05).

### AEBSF Modulates Proteolytic Activity in BALF

**Figure 2:**
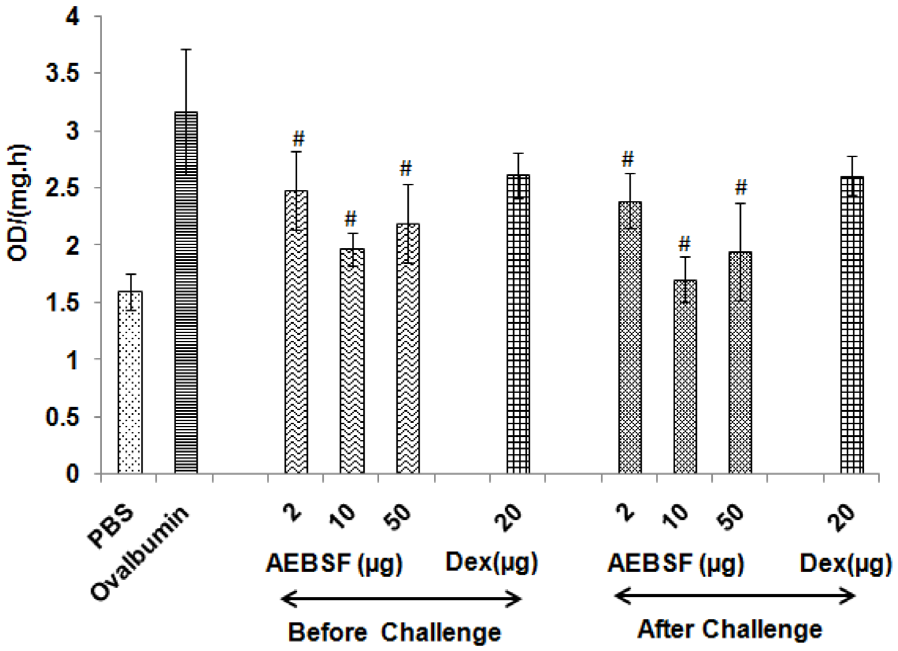
AEBSF reduces proteolytic activity in BALF. Proteolytic activity in term of OD/(mg.h) in ovalbumin challenged and AEBSF/Dex treated mice. Data are presented as mean ± SD of 6 mice per group for one of the two independent mice experiments. #: p<0.05.

Ovalbumin sensitized and challenged mice showed increased protease activity in BALF. AEBSF treatment reduced proteolytic activity significantly, in both before and after challenge treatment groups (Figure 2). However, there was no significant reduction in protease activity in BALF of dexamethasone treated group of mice.

### AEBSF Lowers Mice Serum Antibody Titer

**Figure 3:**
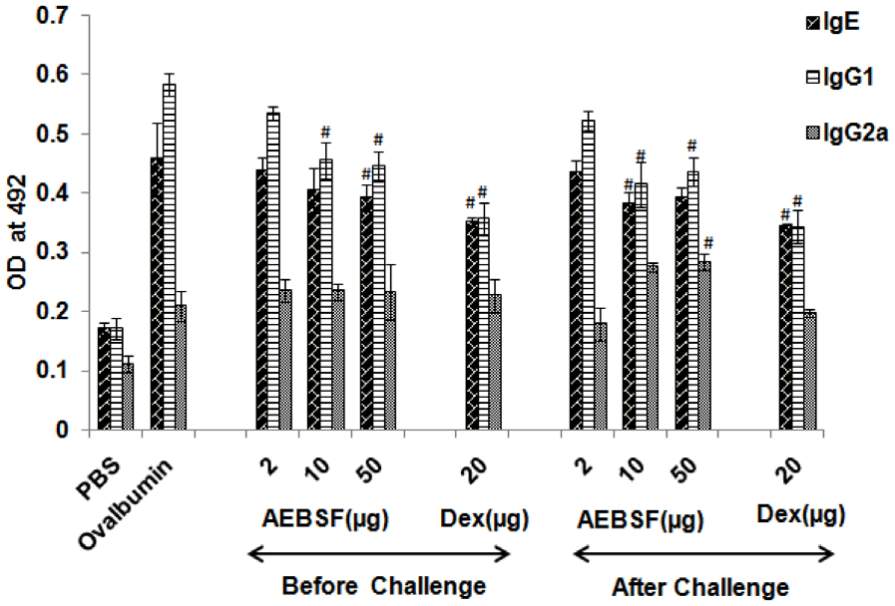
AEBSF/Dex modulates serum immunoglobin level. IgE, IgG1 and IgG2a titer in sera of mice presented as mean ± SD of 6 mice per group for one of the two independent mice experiments. #: p<0.05.

Ovalbumin specific antibody (IgE, IgG1 and IgG2a) levels were measured in the sera of mice by ELISA (Figure 3). There was a reduction in specific IgE but no significant decrease was observed up to 10 µg of AEBSF in before challenge treatment group. Among after challenge groups, 10 µg of AEBSF treated group of mice showed significant decrease in IgE titer in comparison to ovalbumin group mice. IgG1 titer reduced significantly in both, before and after challenge groups of mice at 10 µg and 50 µg doses of AEBSF. Dexamethasone treatment also lowered the IgE and IgG1 titer significantly (p<0.05). The IgG2a level was comparatively higher in before challenge treatment groups than ovalbumin sensitized and PBS treated mice (Figure 3).

### AEBSF Reduces Th2 Cytokines with Enhanced IL- 10 Levels

**Figure 4:**
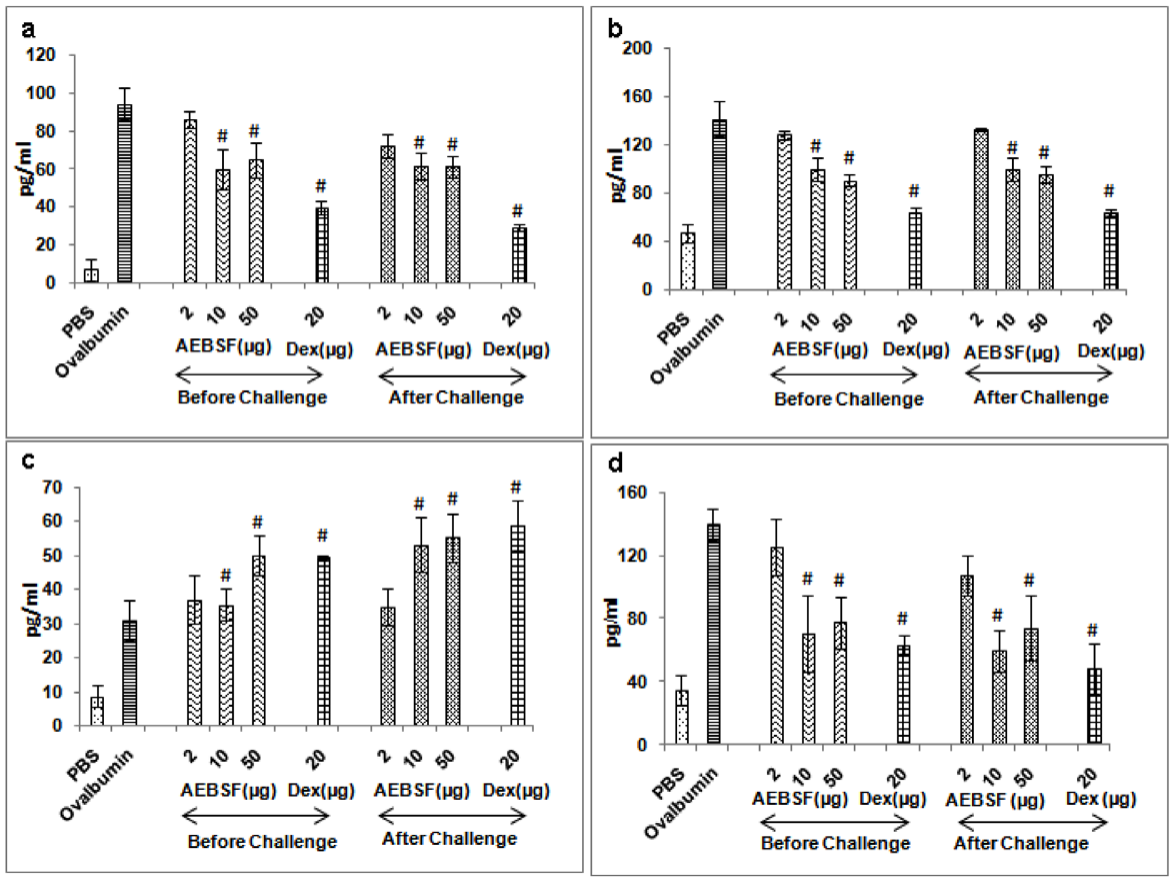
AEBSF/Dex treatment regulates cytokines. (a) IL-4, (b) IL-5, (c) IL-10 and (d) IL-13 level in BALF of ovalbumin challenged and AEBSF/Dex treated groups of mice. Results are in mean ± SD of 6 mice per group for one of the two independent mice experiments. #: p<0.05.

IL-4, IL-5, IL-13 (Th2 cytokines) and IL-10 were analyzed in the BALF samples of mice by ELISA. The absolute values of cytokines in BALF were derived by the reference standard curve. IL-4, IL-5 and IL-13 levels were reduced in before and after ovalbumin challenge treatment groups in a dose dependent manner (Figure 4). IL-10 level was significantly increased with higher dose of AEBSF (50 µg) in both the treatment groups of mice than control. IL-13 level was significantly reduced in mice given 10 µg or 50 µg of AEBSF. Administration of dexamethasone reduced the Th2 cytokines (IL-4, IL-5 and IL-13) significantly (p<0.05) and also up regulated the IL-10 level in both the treatment conditions in mice.

### AEBSF Treatment Suppresses Airway Inflammation

**Figure 5:**
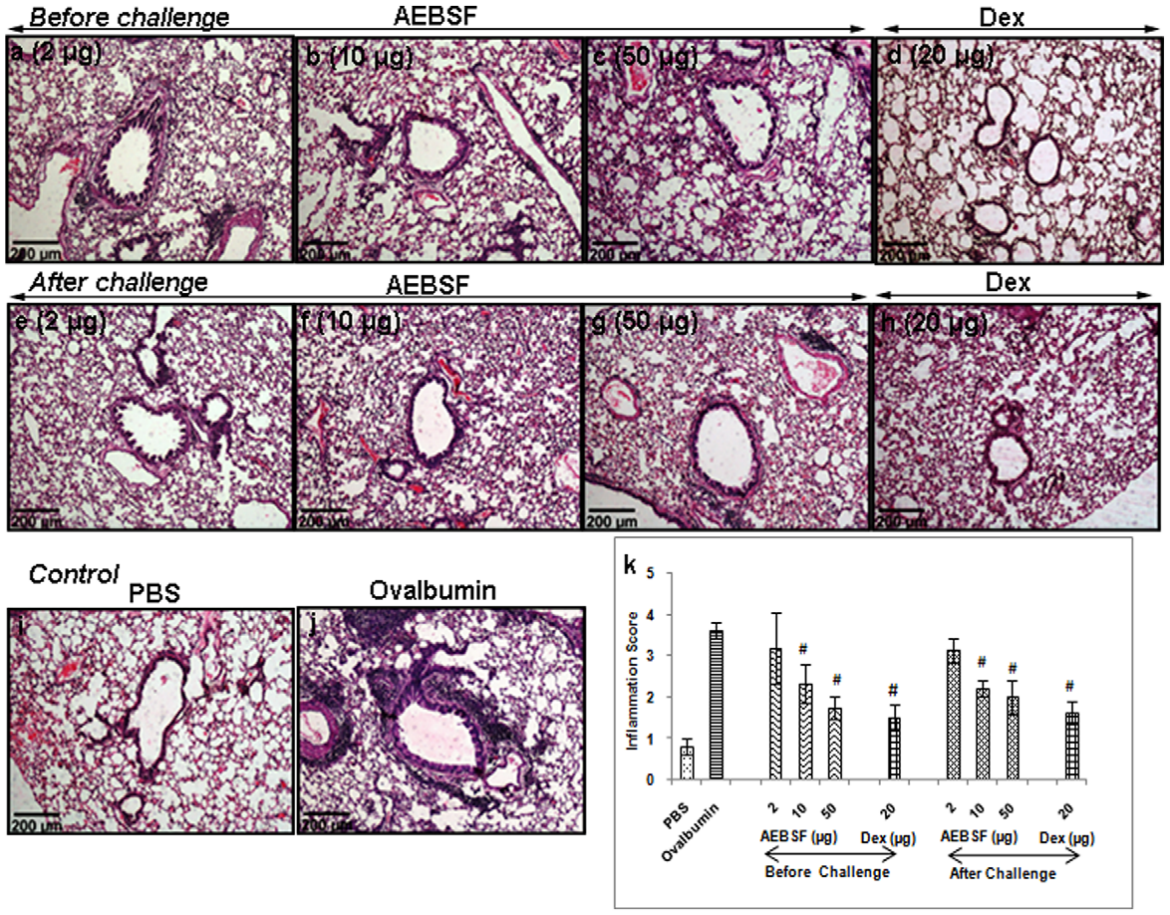
Lung sections of mice stained with H&E (10X). (j) Lung sections indicating cellular infiltration in airways due to ovalbumin sensitization and challenge. (a-h) AEBSF treatment reduces the infiltration of cells. (k) Inflammation score of AEBSF/Dex treated ovalbumin sensitized mice presented as mean ± SD of 6 mice per group for one of the two independent mice experiments. #: p<0.05.

**Figure 6:**
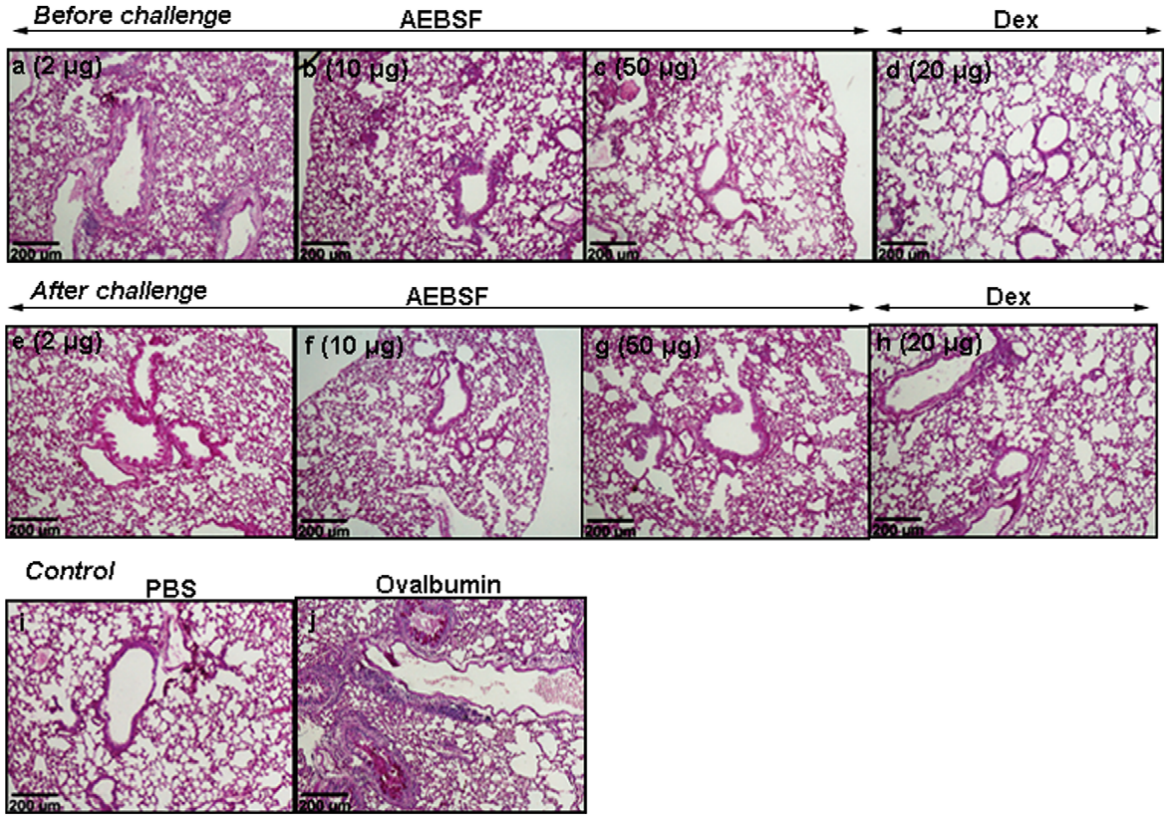
PAS stained lung tissue section **(10X).** (j) The goblet cell hyperpalesia was identified by magenta coloured stain inside the lumen in ovalbumin sensitized mice. (a-h) AEBSF/Dex treatment reduced the goblet cell hyperplasia in ovalbumin sensitized mice.

Lung sections of mice stained with haematoxylin and eosin revealed marked increment in peribronchial and perivascular infiltration of cells in ovalbumin group compared to PBS group. AEBSF treatment reduced the cellular infiltration in ovalbumin induced mouse model in a dose dependent manner in both before and after challenge treatment groups (Figure 5a-h). There was a significant reduction in cellular infiltration with 10 or 50 µg dose of AEBSF, which was further confirmed by a reduced inflammation score both in before and after challenge treatment group of mice in comparison to the ovalbumin group (Figure 5k). PAS stained lung sections revealed that AEBSF treated mice showed reduced goblet cell hyperplasia (Figure 6a-h) as compared to ovalbumin sensitized mice (Figure 6j) indicating low mucus production.

### Reduction in Cysteinyl Leukotrienes Level in BALF by AEBSF Treatment

**Figure 7:**
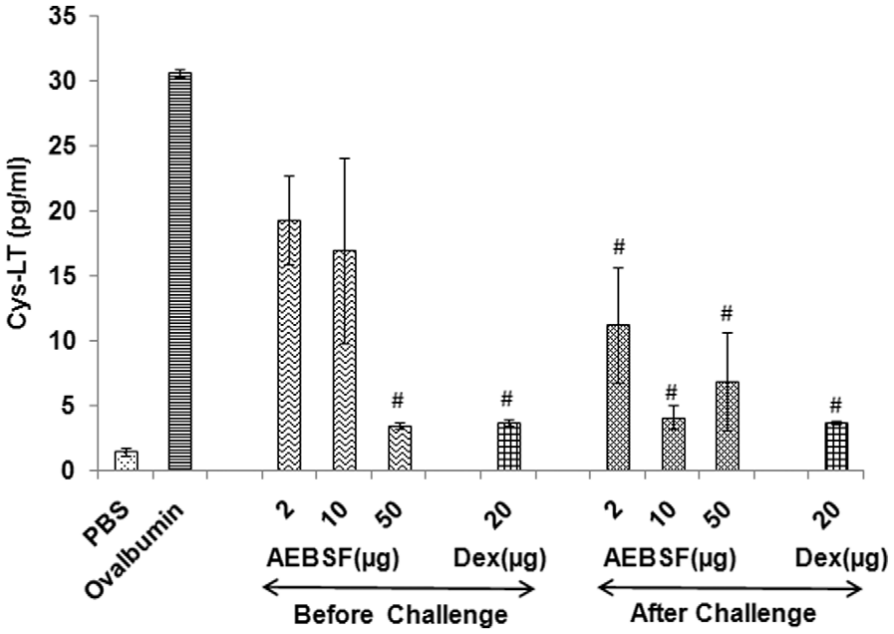
AEBSF/Dex treatment lowers Cys-LT in BALF. Cys-LT levels in BALF of ovalbumin sensitized and AEBSF/Dex treated mice is presented as mean ± SD of 6 mice per group for one of the two independent mice experiments. #: p<0.05.

The Cys-LT level in BALF of mice with different doses of AEBSF treatment and dexamethasone are presented in Figure 7. All the doses of AEBSF (2 to 50 µg) and dexamethasone were effective in reducing Cys-LT level, significantly in after challenge groups whereas only 50 µg dose could significantly reduce the Cys-LT level in before challenge treatment group.

### AEBSF Treatment Reduces 8-isoprostane

**Figure 8:**
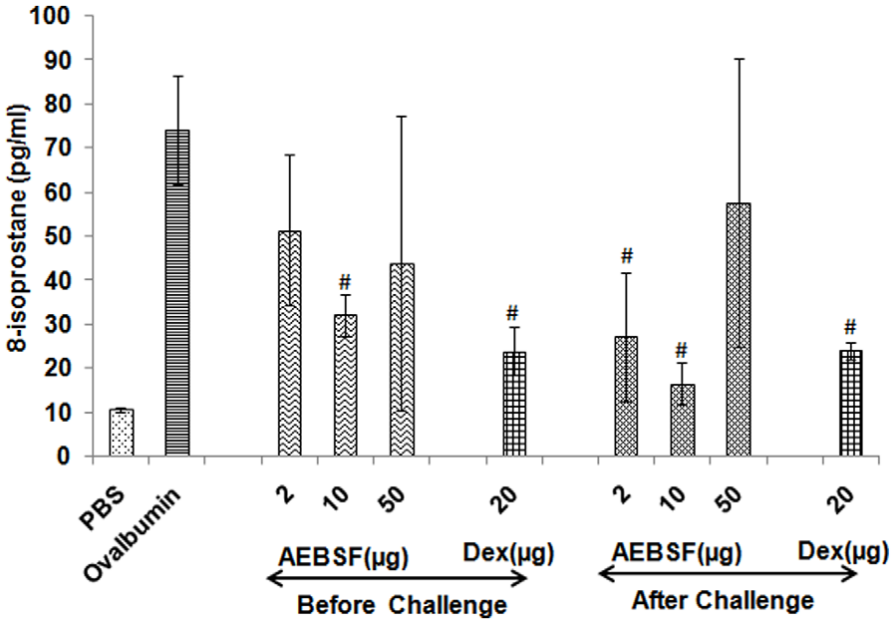
AEBSF/Dex treatment reduces oxidative stress. Oxidative stress was evaluated in terms of 8-isoprostane levels in BALF. Results are presented as mean ± SD of 6 mice per group for one of the two independent mice experiments. #: p<0.05.

To determine the oxidative stress, 8-isoprostane level was measured in BALF by ELISA. Analysis of results revealed that the treatment with 10 µg of AEBSF suppresses the oxidative stress significantly before and after ovalbumin challenge than ovalbumin group mice (Figure 8). Dexamethasone also reduced the oxidative stress marker 8-isoprostane level in BALF significantly.

## Discussion

Asthma is a disease of multifunctional etiology where allergens play a significant role in exacerbation of the symptoms. Most asthma cases are allergic in origin. Studies show that proteases derived from diverse sources are important allergens [20]–[23]. Earlier studies suggest that the protease load in human airways increase significantly following allergen exposure [24] that leads to protease antiprotease imbalance at the respiratory mucosal surfaces [25], [26]. This increase in proteolytic activity contributes to airway pathophysiology as well as airway remodeling associated with the asthma. In the present study, preventive and therapeutic effects of a protease inhibitor, AEBSF was investigated in ovalbumin induced mouse model of allergic airway disease.

Previously, a tryptase inhibitor APC366 reduced IgE and non-IgE mediated histamine release from mast cells [27], [28]. Tryptase inhibitor MOL 6131 inhibited release of Th2 cytokines in mouse model [29]. Further, serine protease inhibitors-nefamostat mesilate and gabexate mesilate suppressed Der p induced allergic response, when given during sensitization and after challenge [30]. In the present study, AEBSF treatment was given in sensitized mice, which is clinically relevant for patients. Mice were given intranasal treatment to limit the effect of protease inhibitor on affected tissue and to avoid possible side effects. The dose of AEBSF treatment in the present study was determined by preliminary experiment.

In the present study, repeated exposure with ovalbumin induced Th2 milieu in mice with subsequent increase in serum IgE and IgG1 and cytokines like IL-4 IL-5 and IL-13 in BALF. AEBSF treatment showed significant reduction in IgE with higher dose in both before and after antigen challenge treatment groups. IgG1 titer also reduced significantly in both treatment groups of mice at 10 µg and 50 µg dose of AEBSF. IL-4, IL-5 and IL-13 levels decreased on treatment with AEBSF, whereas IL-10 level increased significantly with 50 µg dose of AEBSF. Studies with SLPI [31], nefamostat mesilate and gabexate mesilate induced IL-10 that has suppressed allergen induced airway inflammation [32].

IgE cross linking with allergen degranulates mast cells and basophils releasing tryptase and proinflammatory mediators. Tryptase is a known inducer of eosinophil and neutrophil infiltration [33]. These cells are involved in late phase allergic reactions and produce peroxidase that degrades self tissue and increases oxidative burden [34]. The present data demonstrate increased proteolytic activity in BALF of ovalbumin challenged mice and AEBSF treatment reduced cellular infiltration and proteolytic/peroxidase activity.

Allergen activates inflammatory cells to release inflammatory mediators including cysteinyl leukotrienes [35] that induces bronchoconstriction [36] and mucus production [37]. AEBSF treatment reduced (50 µg before/2–50 µg after challenge treatment) the Cys-LT level in BALF indicating its therapeutic potential. Histology of lung tissue also showed decreased cellular infiltration in AEBSF treated mice. This led to significant reduction (p<0.05) in inflammation score compared to ovalbumin control group. This is in accordance with an earlier study where nefamostat mesilate and gabexate mesilate were used as inhibitors [38].

In asthma, the balance between oxidants and antioxidants in the airways is altered, leading to the excessive production of ROS [39]. ROS enhances the production of lipid derivatives such as 8-isoprostane which induces contraction in airway smooth muscle cells *in vitro* [40]. AEBSF treatment in before challenge group (10 µg) and in after challenge group (2 µg and 10 µg) showed significant decrease in 8-isoprostane level in BALF. This is in agreement with the previous studies showing inhibition of NADPH oxidase, a key enzyme that catalyses the ROS production in phagocytes by AEBSF [41]. Besides, AEBSF is known to induce the expression of heme oxigenase I via protein kinase B pathway. Heme oxigenase degrades the pro-oxidant heme and produces anti-oxidant bilirubin which further provides cellular protection against oxidative stress [42]. AEBSF inhibits NADPH oxidase and reduces superoxide production [43]. It also inhibits the invasion of parasites *in vitro* [44] and attenuate vasogenic brain edema formation in newborn pigs [45].

Although both the treatment protocols were effective in reducing airway inflammation, therapeutic treatment reduced inflammation, Cys-LT and oxidative stress more effectively than the prophylactic treatment. AEBSF reduced the IL-5 levels in dose dependent manner in both protocols.

Current therapies for asthma mainly include use of corticosteroids, and short- and long-acting β2 adrenergic receptor agonists, based on the severity of the symptoms. Although these therapies are effective in providing symptomatic relief, none of the therapies are curative. Also the use of oral steroids for long terms showed undesirable side effects. Serine protease inhibitors are regarded as potential therapeutic option to reduce the use of steroid in airway inflammatory diseases. In conclusion, AEBSF significantly reduces allergic airway inflammation and has potential for adjunct therapy in allergic diseases including asthma.

## Materials and Methods

### Animal and Ethics

Female BALB/c mice of 6–8 weeks weighing 18–22 grams were procured from Central Drug Research Institute, Lucknow (India). Mice were housed for 2 weeks to get acclimatized to experimental conditions. Standard chow diet (*ad labitum*) and purified water were provided to mice, kept at 40–70% humidity with 12 hour controlled light: dark cycle. All the experimental procedures were carried out in the morning to minimize the effect of circadian rhythm. The animal experiment was repeated twice and the results of one of the two independent experiments are presented. The study protocol was approved by animal ethics committee of Institute of Genomics and Integrative Biology, Delhi.

### Immunization and Treatment Protocol

Mice were divided into 10 groups randomly, containing 6 mice each and sensitized on day 0 and 14 by *i.p.* injection of 100 µg ovalbumin adsorbed on 2 mg alum. They were challenged with 2 µg of ovalbumin on day 25, 26 and 27 by *i.n*. route. Mice were treated with 2, 10 and 50 µg of AEBSF (Sigma) *i.n.* 1 hour before or after ovalbumin challenge in groups. Dexamethasone (20 µg) was given to the mice 1 hour before or after ovalbumin challenge through *i.n* route as treatment control. Intranasal treatment was given by anesthetizing mice with 3% isoflurane. One group of mice was treated with dexamethasone (20 µg) 1 hour before challenge and another group 1 h after challenge. One group (negative control) of mice were sensitized, challenged and treated with PBS. Similarly a group of mice were sensitized/challenged with ovalbumin and treated with PBS.

### Sample Collection

Mice were euthanized by *i.p*. injection of sodium thiopentone (100 mg/kg) on day 29. BALF was collected from each mouse by instillation of 0.5 ml of chilled PBS, thrice into the lung. BALF was centrifuged at 400 g for 10 min at 4 °C and supernatant was used for analysis of cytokines, leukotrines, 8-isoprostane and eosinophil peroxidase activity. Bronchoalveolar lavage cell pellet was used to determine cellular infiltration. Blood was collected, allowed to clot and sera were separated by centrifugation at 400 g for 10 min. Lungs were incised from thoracic cavity of mice and fixed with 10% neutral-buffered formalin for histopathology.

### Eosinophil Count and Eosinophil Peroxidase Activity in BALF

The bronchoalveolar lavage cell pellet was resuspended in PBS and total cell count per ml in BALF was determined using trypan blue (Sigma Aldrich Co., St. Luis, USA) and haemocytometer **(**Neuber’s), under light microscope. Eosinophils were counted by staining BALF cell smear with Leishman stain. Percentage of eosinophils and neutrophils was determined by counting minimum of 200 cells. Absolute numbers of eosinophils and neutrophils in per ml of BALF were calculated by total cell counts and percentage value [46].

Eosinophil peroxidase activity was measured by spectrophotometric method. Briefly, 100 µl of BALF supernatant from each sample was taken in microtitter plate in duplicates (Nunc-immuno, Denmark). One hundred microliter of substrate solution containing 0.1 mM o-phenylene-diamine-dihydrochloride, 0.1% Triton X-100 and 1 mM hydrogen peroxide in 0.05 M Tris-HCl was added in each well and incubated for 30 min at 37°C. Reaction was stopped by adding 50 µl of 4 M sulphuric acid. Absorbance was measured at 492 nm using microplate spectrophotometer reader (Bio-Rad Laboratories Ltd., UK). EPO activity of BALF supernatant was depicted in the term of OD per min per mg of protein [47].

### Proteolytic Activity in BALF of Mice

To evaluate proteolytic activity in BALF, azo dye impregnated collagen (Sigma Aldrich) was used as a substrate. Ten milligram of substrate in 1 ml Tris-HCl buffer (pH 6.5) was incubated with 20 µl of BALF at 37°C. After 60 min incubation at constant shaking of 200 rpm, 4% trichloroacetic acid was added to stop the reaction. Mixture was centrifuged and absorbance was measured at 520 nm [48]. One unit of specific activity was defined as 1.00 changes in absorbance per microgram of protein per hour [49].

### Measurement of Ovalbumin Specific Antibodies in Sera of Mice

Relative concentrations of ovalbumin specific IgE, IgG1 and IgG2a were measured in serum samples of mice by indirect ELISA [46]. Briefly, microtiter plates (Nunc-Immuno, Denmark) were coated with 500 ng ovalbumin/well in 100 µl of 0.1 M carbonate buffer (pH 9.6) and incubated overnight at 4 °C. After washing with PBS, plates were blocked with 3% defatted milk for 3 h at 37 °C. Sera samples were diluted in PBS 1∶10, 1∶50 and 1∶50 v/v for IgE, IgG1 and IgG2a, respectively and used in triplicates for estimation. In each well, 100 µl of diluted sera was added and the plates were incubated overnight at 4°C. Unbound antibodies were removed by washing the plates with PBST (0.05% Tween-20 in PBS) and subsequently incubated for 3 h at 37°C with anti-mouse IgG1-peroxidase or anti-mouse IgG2a-peroxidase (1∶1000 PBS; BD Pharmingen, San Diego, CA, USA). For IgE estimation, the plate was incubated with biotinylated anti-mouse IgE (2 µg/mL, BD Pharmingen) at 25°C for 90 min followed by streptavidin-peroxidase (1∶1000; BD Pharmingen) for 30 min. After washing, plates were developed using o-phenylenediamine and absorbance was read at 492 nm.

### Estimation of Cytokines in BALF

Cytokines IL-4, IL-5, IL-10 (BD Pharmingen, USA and R & D, MN, USA) and IL-13 (R&D Systems Minneapolis, MN USA) were determined in BALF by ELISA following manufacturer’s instructions. Briefly, 100 µl capture antibody (1∶250 v/v) for each cytokine was coated separately in microtiter plates in carbonate buffer, pH 9.6 (in phosphate buffer, pH 6.5 for IL-10 capture antibody) and incubated for 12 hour at 4°C. After washing with PBS, plates were blocked with assay diluent (10% foetal bovine serum in PBS) at 25°C for 1 h. Plates were washed with PBS and 100 µl of standards (seven serial dilutions) and undiluted BALF samples were added to the wells in duplicates. After incubation at 25°C for 2 h, the plates were washed and incubated with biotinylated detector antibody labeled with avidin-horse radish peroxidise (HRP) at 25°C for 1 h. After washing, 100 µ1of tetramethylbenzidine (Sigma Aldrich) substrate solution was added in each well and developed by incubating at 37°C in dark. Reaction was stopped by addition of 4 N H_2_SO_4_ and absorbance was measured at 450******nm (wavelength correction at 570 nm) using microplate reader (Bio-Rad).

### Cysteinyl Leukotrienes Levels in BALF

Cysteinyl leukotriene (Cys-LT) levels were determined in BALF using enzyme immunoassay kit (Cayman Chemical Co. MI, USA). Briefly, 50 µl of standards (8 serial dilutions) or BALF was added per well of microtiter plates in duplicates. To this, 50 µl of tracer and 50 µl of Cys-LT antiserum was added and incubated at 25°C for 18 h. Following washing with buffer, plate was developed by adding Ellman’s reagent and incubating in dark at 25°C for 1.5 h. Absorbance was read periodically at 420 nm.

### Measurement of Oxidative Stress

The level of 8-isoprostane in BALF was measured using enzyme immunoassay kit (Cayman Chemical) following the manufacturer’s instructions. Briefly, standards in different dilutions and BALF samples were mixed (50 µl/well) with 50 µl of 8-isoprostane tracer and 50 µl of 8-isoprostane antiserum and then incubated at 4°C for 18 h. After washing with wash buffer, 100 µl of substrate solution was added to each well and incubated in dark at 25°C for 90 min with gentle shaking. Absorbance was read periodically at 420 nm until the absorbance of maximum binding wells reached a minimum of 0.3 A.U (Absorbance unit).

### Histopathology

The lungs were embedded in paraffin and sections of 4 µm thickness were cut and stained with haematoxylin and eosin. Histological evaluation was made under microscope (10X) and scored on the basis of cellular infiltration and narrowing of airway lumen. Using gridline the total square area around the lumen of airways has been counted and the percentage of square falling under the inflamed area had been evaluated and regarded as percentage affected area. And the scoring was made from 1–5. ‘one’ inflammatory reaction affecting <20% of the airways, ‘two’ as 20–40% of the airways affected, ‘three’, 40–60%, ‘four’, 60–80% and ‘five’, >80% of the airways affected. Beside this figure of PAS stained lung section is included in manuscript.

### Statistical Analysis

Statistical analysis of results was done by using GraphPad Prism and GraphPad Instat software (GraphPad Software, San Diego, CA, USA). The statistically significant difference was determined using one way ANOVA followed by Dunnett’s multiple comparison tests between ovalbumin challenged and PBS treated and AEBSF treated mice groups. The p value <0.05 was considered as significant [46].
